# Supplementary material for: Multi‐dataset identification of innovative feature genes and molecular mechanisms in keratoconus
Source: J Cell Mol Med. 2024 Sep 19;28(18):e70079. doi: 10.1111/jcmm.70079 (PMC11412914; doi:10.1111/jcmm.70079)
Supplement: Supplementary file 2 — Material S2. [file JCMM-28-e70079-s002.zip › 7.GSEA analysis/3.DBNDD1/KEGG_GLYCOSAMINOGLYCAN_BIOSYNTHESIS_CHONDROITIN_SULFATE.html]

Details for gene set KEGG\_GLYCOSAMINOGLYCAN\_BIOSYNTHESIS\_CHONDROITIN\_SULFATE[GSEA]

|  || Dataset | DBNDD1.group.cls#h\_versus\_l |
| Phenotype | group.cls#h\_versus\_l |
| Upregulated in class | l |
| GeneSet | KEGG\_GLYCOSAMINOGLYCAN\_BIOSYNTHESIS\_CHONDROITIN\_SULFATE |
| Enrichment Score (ES) | -0.50252354 |
| Normalized Enrichment Score (NES) | -1.3676974 |
| Nominal p-value | 0.115913555 |
| FDR q-value | 0.7556042 |
| FWER p-Value | 0.919 |
Table: GSEA Results Summary

  

Fig 1: Enrichment plot: KEGG\_GLYCOSAMINOGLYCAN\_BIOSYNTHESIS\_CHONDROITIN\_SULFATE      
 Profile of the Running ES Score & Positions of GeneSet Members on the Rank Ordered List

  

| SYMBOL | TITLE | RANK IN GENE LIST | RANK METRIC SCORE | RUNNING ES | CORE ENRICHMENT || 1 | CHSY3 | na | 6032 | 0.150 | -0.1017 | No |
| 2 | XYLT1 | na | 11667 | 0.050 | -0.2398 | No |
| 3 | CHST12 | na | 12816 | 0.040 | -0.2538 | No |
| 4 | CHST3 | na | 14725 | 0.025 | -0.2968 | No |
| 5 | B3GALT6 | na | 14781 | 0.024 | -0.2866 | No |
| 6 | B4GALT7 | na | 17058 | 0.009 | -0.3479 | No |
| 7 | CSGALNACT1 | na | 17492 | 0.006 | -0.3573 | No |
| 8 | CHSY1 | na | 19561 | -0.009 | -0.4125 | No |
| 9 | CHPF | na | 20139 | -0.014 | -0.4223 | No |
| 10 | B3GAT1 | na | 21566 | -0.028 | -0.4501 | No |
| 11 | XYLT2 | na | 21761 | -0.030 | -0.4414 | No |
| 12 | B3GAT3 | na | 21930 | -0.031 | -0.4312 | No |
| 13 | DSE | na | 24236 | -0.060 | -0.4689 | Yes |
| 14 | CHST13 | na | 24432 | -0.062 | -0.4446 | Yes |
| 15 | CHPF2 | na | 24676 | -0.066 | -0.4197 | Yes |
| 16 | CHST11 | na | 27550 | -0.120 | -0.4448 | Yes |
| 17 | CHST7 | na | 29049 | -0.158 | -0.4116 | Yes |
| 18 | B3GAT2 | na | 29281 | -0.165 | -0.3386 | Yes |
| 19 | CSGALNACT2 | na | 29891 | -0.185 | -0.2669 | Yes |
| 20 | UST | na | 30136 | -0.192 | -0.1813 | Yes |
| 21 | CHST15 | na | 30279 | -0.197 | -0.0906 | Yes |
| 22 | CHST14 | na | 34165 | -0.454 | 0.0162 | Yes |
Table: GSEA details [plain text format]

  

Fig 2: KEGG\_GLYCOSAMINOGLYCAN\_BIOSYNTHESIS\_CHONDROITIN\_SULFATE      
 Blue-Pink O' Gram in the Space of the Analyzed GeneSet

  

Fig 3: KEGG\_GLYCOSAMINOGLYCAN\_BIOSYNTHESIS\_CHONDROITIN\_SULFATE: Random ES distribution      
 Gene set null distribution of ES for **KEGG\_GLYCOSAMINOGLYCAN\_BIOSYNTHESIS\_CHONDROITIN\_SULFATE**
